# To the Dental Profession

**Published:** 1877-02

**Authors:** 


### ARTICLE VI.

### *To The Dental Profession.*

We the undersigned members of a Committee appointed by a general meeting of the Dentists of New York and Brooklyn held at the New York College Dentistry, Nov. 11th, 1876, adopt this method of calling the earnest attention of the profession to a new phase of an old but important subject, and of accomplishing at the same time the object for which we were appointed. We have learned that in the defence of certain cases in which suits have been instituted by the Goodyear Dental Vulcanite Co., against Dentists for infringement of the Cummings' patent, steps have been taken to lay before the Courts a line of defence and system of evidence different from those of any other case heretofore heard.

We have satisfied ourselves by proper measures that the facts and testimony to be presented in this line of defence are of such character, as to leave little if any doubt of their competency to deliver the Dental Profession from the onerous and unjust taxation to which it has been so long subjected; in other words that it is in our power now to prove the illegality and invalidity of the Cummings' patent. We say this with a full knowledge and appreciation of the fact of former efforts and failures.

With these firm convictions we not only ask but urge the Dentists of New York as well as other States, to contribute their efforts and means both as Societies and as individuals, to aid and sustain those who are working in behalf of the interests and just rights of the profession of the whole country; for every member of it is interested either directly or indirectly in the issue of these suits.

We make this appeal because where such large interests are involved, and the testimony of so many witnesses in different localities is to be taken, the expense must be necessarily large in proportion; but this expense though large can be made to bear lightly by its division among many. Therefore if each Dentist who can be reached, [all of course cannot,] would contribute a small sum, the amount needed could be obtained readily, and without inconvenience to any. If the means are furnished the work will be done by earnest and honest men.

The defence in this instance is being conducted carefully, and with every effort to make it much less expensive than former ones. We feel a deep interest in this cause, we are doing all we can for it, and we urge every member of the profession to whose notice this may come, to subscribe such amount as he may see fit, and forward it to the Treasurer of this Committee, or to Dr. R. Finley Hunt, 1806 H. Street, Washington, D. C.; a receipt will be sent by return mail.

Where Societies are already organized we suggest that they take action in this matter. Where there are none, a temporary organization be formed so that its Officers or Committee can solicit, receive and forward subscriptions.

Dr. Wm. H. Dwinelle, Chairman, 27 W. 34th St., N. Y.
Dr. Wm. H. Allen, Treasurer, 18 West 11th Street, N. Y.
Dr. Norman W. Kingsley,
Dr. Frank Abbott,
Dr. C. A. Woodward,
A. N. Chapman, Brooklyn.

Since the above was put in type the Supreme Court of the United States has announced its decision affirming that of the U. S. Circuit Court for the District of Massachusetts, in favor of the Goodyear Dental Vulcanite Co., vs. Daniel H. Smith, three of the Justices dissenting.

This decision will not govern the cases which we are now defending, as our line of defence is almost entirely different from the one pursued in that case.

This decision was anticipated by many of those who were aware of the line of defence in the Smith case. Therefore we are not altogether disappointed, nor are we at all swerved by it from our determination to continue our defence to the end, with the fullest confidence of success.

